# Role of Tau as a Microtubule-Associated Protein: Structural and Functional Aspects

**DOI:** 10.3389/fnagi.2019.00204

**Published:** 2019-08-07

**Authors:** Pascale Barbier, Orgeta Zejneli, Marlène Martinho, Alessia Lasorsa, Valérie Belle, Caroline Smet-Nocca, Philipp O. Tsvetkov, François Devred, Isabelle Landrieu

**Affiliations:** ^1^Fac Pharm, Aix Marseille Univ., Centre National de la Recherche Scientifique (CNRS), Inst Neurophysiopathol (INP), Fac Pharm, Marseille, France; ^2^Univ. Lille, Centre National de la Recherche Scientifique (CNRS), UMR 8576, Unité de Glycobiologie Structurale et Fonctionnelle (UGSF), Lille, France; ^3^Univ. Lille, Institut National de la Santé et de la Recherche Médicale (INSERM), CHU-Lille, UMR-S 1172, Centre de Recherche Jean-Pierre AUBERT (JPArc), Lille, France; ^4^ Aix Marseille Univ., Centre National de la Recherche Scientifique (CNRS), UMR 7281, Bioénergétique et Ingénierie des Protéines (BIP), Marseille, France

**Keywords:** post-translational modifications, biophysical methods, Alzheimer’s disease, intrinsically disordered proteins, neurodegenerative diseases

## Abstract

Microtubules (MTs) play a fundamental role in many vital processes such as cell division and neuronal activity. They are key structural and functional elements in axons, supporting neurite differentiation and growth, as well as transporting motor proteins along the axons, which use MTs as support tracks. Tau is a stabilizing MT associated protein, whose functions are mainly regulated by phosphorylation. A disruption of the MT network, which might be caused by Tau loss of function, is observed in a group of related diseases called tauopathies, which includes Alzheimer’s disease (AD). Tau is found hyperphosphorylated in AD, which might account for its loss of MT stabilizing capacity. Since destabilization of MTs after dissociation of Tau could contribute to toxicity in neurodegenerative diseases, a molecular understanding of this interaction and its regulation is essential.

## Introduction

Tau is a microtubule-associated protein (MAP; Weingarten et al., 1975; Witman et al., 1976) that is abundant in the axons of neurons where it stabilizes microtubule (MT) bundles (Binder et al., 1985; Black et al., 1996). Together with other destabilizing MAPs, such as stathmin, Tau plays a central role in MT dynamics by regulating assembly, dynamic behavior and the spatial organization of MTs. Tau and other MAPs that bind to MTs are tightly regulated by a number of factors, including post-translational modifications (PTMs), to ensure the appropriate dynamics of the system (Lindwall and Cole, 1984; Mandelkow et al., 1995; Ramkumar et al., 2018). However, the exact mechanism of assembly and stabilization of MTs by Tau remains challenging to characterize due to the inherent dynamics of the system and the disordered nature of Tau. The number of approaches that have been conducted so far to gain knowledge on this particular interaction is striking (Devred et al., 2010; Nouar et al., 2013; Di Maio et al., 2014; Tsvetkov et al., 2019). Tau is probably the most studied MAP because of its implication in a group of neurodegenerative diseases called tauopathies, associated with Tau aggregation into intraneuronal deposits (Brion et al., 1986), such as frontotemporal dementia (FTD), Alzheimer’s disease (AD) and progressive supranuclear palsy.

Tau binding to MTs, as well as its propensity to aggregate, are affected by Tau mutations and by Tau PTMs, in particular, its hyper-phosphorylation (Alonso et al., 1994; LeBoeuf et al., 2008). These pathologically modified Tau molecules perturb MT function and axonal transport, contributing to neurodegeneration. Indeed, a reduction of MT density and fast axonal transport is observed in transgenic AD mouse models that exhibit hyperphosphorylated Tau inclusions in neurons (Cash et al., 2003). Furthermore, Tau mutations carried by FTD patients cause MT-mediated deformation of the nucleus, which in turn results in perturbation of the nucleocytoplasmic transport (Paonessa et al., 2019). In addition, mislocalized Tau in neurons of Tau-overexpressing transgenic mouse brain and of human AD brain directly interacts with the nucleoporins of the nuclear pore complex. This interaction disrupts the nuclear pore functions of nucleocytoplasmic transport and might contribute to Tau-related neurotoxicity (Eftekharzadeh et al., 2018).

Efforts for clinical intervention in the AD field have so far mainly focused on blocking the formation of extracellular amyloid β deposits, another type of aggregates observed in the brain of AD patients (Cummings et al., 2017). However, in the last several years, there has been an increase in studies aimed at the therapeutic targeting of Tau. An improved understanding of Tau functions could lead to the development of new strategies of therapeutic interventions (Mudher et al., 2017; Jadhav et al., 2019). This emphasizes the need to understand the finer details of structural and functional aspects of Tau/MTs interaction.

## Tubulin and MTs

**Figure 1:**
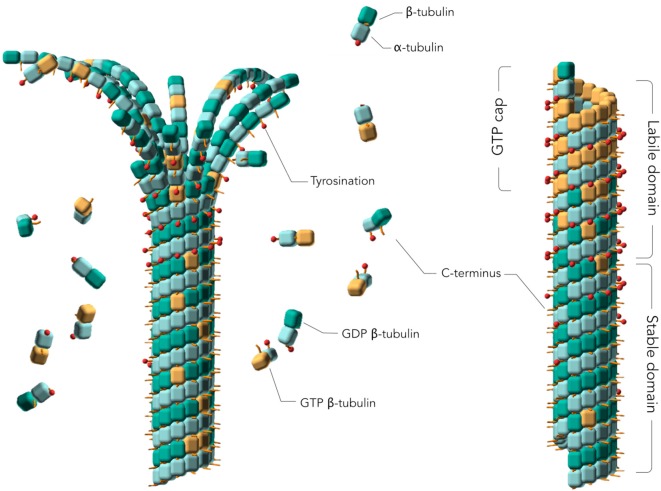
Assembly of tubulins into Microtubules (MTs). Models of a depolymerizing (left) and polymerized (right) MTs. The guanosine 5′ triphosphate (GTP) cap or labile domain is composed mostly of tyrosinated GTP-tubulin. The stable domain is mostly composed of detyrosinated guanosine 5′ diphosphate (GDP)-tubulin. β-tubulin subunits are represented as orange cubes in their GTP-bound states and green cubes in their GDP-bound states. α-tubulin subunits are represented as blue cubes. Red sticks planted on the cubes represent the C-terminal tail of α- and β-tubulin subunits. Tyrosination is represented by a red dot on the C-terminal tail. Two MT regions are distinguished: a labile MT region mostly composed of tyrosinated GTP-tubulins, including the GTP cap, and a stable MT region mostly consisting of assembled detyrosinated GDP-tubulins. MT depolymerization is characterized by curled protofilaments at MT ends (*left*).

MTs are hollow cylinders composed of parallel protofilaments of α and β tubulin subunits (α/β tubulin heterodimer, here named tubulin) assembled head-to-tail, which form a pseudo helical lattice (Amos and Schlieper, 2005; Figure 1). During MT formation, tubulins self-assemble in their guanosine 5′ triphosphate (GTP)-bound state to form a sheet that closes into a 25 nm outside diameter tube (Chrétien et al., 1995). Whereas the MT core or lattice is constituted of guanosine 5′ diphosphate (GDP)-tubulin, a GTP cap forms at MT ends because of the delay between assembly and GTP hydrolysis at the tubulin inter-dimer interface (Carlier et al., 1989; Caplow and Shanks, 1990; Bowne-Anderson et al., 2015; Baas et al., 2016). This cap has been proposed to stabilize growing MTs since its loss induces MT depolymerization, with characteristic curled protofilaments at MT ends (Chrétien et al., 1995). MTs constantly undergo phases of assembly and disassembly in a process called “dynamic instability” (Mitchison and Kirschner, 1984). Due to these intrinsic dynamics of the system, MTs have been difficult to study from a structural point of view. During the past decades, numerous molecular models were used to simulate the mechanism of MT formation, resulting in a more nuanced model than the original GTP-cap one, which now accounts for the diverse regulatory activities of MAPs. In this model named “coupled-random model,” GTP hydrolysis occurs with a constant rate for any tubulin in the MT lattice, except for the dimers at the very tip (Bowne-Anderson et al., 2015). GDP-tubulin does not assemble into MTs but forms double rings (Howard and Timasheff, 1986). It was thus originally considered that GTP would allosterically induce a straight conformation of tubulin capable of MT assembly and that GDP would induce a curved conformation favoring disassembly (Melki et al., 1989). It is now generally accepted that GTP-tubulin in solution is curved similarly to GDP-tubulin, based on the MT models derived from electron microscopy (EM) data, up to about 3.5 Å resolution (Zhang R. et al., 2015) and from X-ray crystallography up to 2 Å resolution (Nawrotek et al., 2011; Pecqueur et al., 2012) together with biochemical evidence (Barbier et al., 2010). Tubulin only straightens upon incorporation into MTs (Alushin et al., 2014). In addition to the GDP/GTP dual nature of tubulin, PTMs of tubulin, such as tyrosination and acetylation, modulate MT stability (Baas et al., 2016). In neurons, two MT regions are distinguished (Figure 1): a labile MT region mostly composed of tyrosinated and deacetylated GTP-tubulin, and a stable MT region (or lattice) mostly consisting of assembled detyrosinated and acetylated GDP-tubulin (Baas et al., 2016). These findings together with other recent studies of tubulin modifications—including phosphorylation, polyglycylation, deglutamylation and polyglutamylation—highlight the importance of these PTMs in regulating the different functions of MTs, generating a “tubulin code” (Gadadhar et al., 2017). Indeed, a defect of the deglutamylase CCP1, the enzyme responsible for tubulin deglutamylation, causes infantile-onset neurodegeneration (Shashi et al., 2018), while an excessive polyglutamylation of tubulin reduces the efficiency of neuronal transport in cultured hippocampal neurons (Magiera et al., 2018). Moreover, a default of acetylated tubulin is linked to abnormal axonal transport in several neurodegenerative diseases (Dompierre et al., 2007; Godena et al., 2014; Guo et al., 2017). Altogether, these studies suggest that enzymes responsible for tubulin PTMs could be promising therapeutic targets.

## Tau Protein

**Figure 2:**
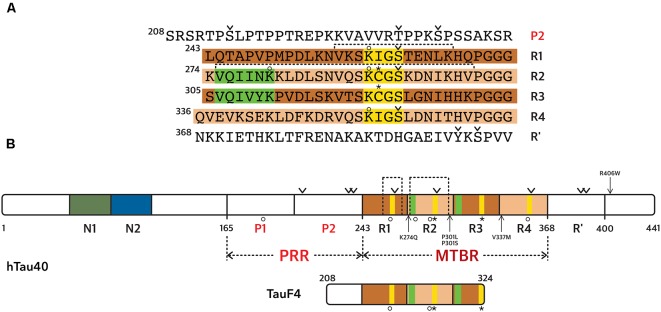
Tau protein sequence and domain organization. The sequence numbering is according to the longest Tau isoform (441 amino acid residues). **(A)** Amino acid sequence of the microtubule binding region (MTBR) and flanking regions, P2 in the PRR and R’ in the C-terminal domain. **(B)** General scheme of the full-length Tau protein and of TauF4 fragment. **(A,B)** The MTBR region of Tau consists of four partially repeated sequences, R1 to R4, highlighted in dark and light brown. PHF6* and PHF6 hexapeptides, in R1 and R2 repeats respectively, are highlighted in green and the KXGS motifs in yellow. Phosphorylation sites mentioned in the text are indicated with a v sign, cysteine residues with a star sign and acetylation with a circle. Segments of R1 and R2 shown in Figure 3 are indicated with dashed lines.

There are two major MAPs present in cells from the central nervous system, MAP2 and Tau. The expression of one or the other isoform is regulated during development, and their localizations differ. Tau is mainly found in the axonal compartment, while MAP2 is expressed specifically in cell bodies and dendrites (Melková et al., 2019). Both proteins exist as alternatively spliced isoforms, with some high-molecular weight isoforms for MAP2 (MAP2a and MAP2b). There are six isoforms of Tau protein present in the central nervous system (ranging from 352 to 441 amino acid residues), resulting from mRNA alternative splicing of a single gene (Goedert et al., 1989; Himmler et al., 1989). Tau is divided into functional domains (numbering according to the longest isoform, Figure 2): an N-terminal projection domain Tau(1–165), a proline-rich region Tau(166–242) or PRR, a MT binding region Tau(243–367) or microtubule binding region (MTBR) and a C-terminal domain Tau(368–441). The MTBR consists of four partially repeated sequences R1(243–273), R2(274–304), R3(305–335), and R4(336–367; Figure 2). The isoforms differ by the presence, or not, of one/two insert(s) in the N-terminal domain (N1 and/or N2 presence or not), and three or four repeat sequences in the MTBR (R2 presence or not). Even though Tau is an intrinsically disordered protein (IDP), it has a tendency to form local secondary structures, in particular, β-strands in the MTBR and polyproline II helices in the PRR (Mukrasch et al., 2009; Sibille et al., 2012). It has been proposed that Tau N- and C-terminal domains fold back on the central PRR and MTBR domains due to contacts between the N- and C-termini of the protein (Jeganathan et al., 2006; Mukrasch et al., 2009). This model was initially built using distance measurements based on Förster Resonance Energy Transfer (FRET) detection between pairs of fluorophores attached to Tau (Jeganathan et al., 2006). Moreover, measurements of paramagnetic relaxation enhancement (PRE) by nuclear magnetic resonance spectroscopy (NMR) using paramagnetic centers attached at different positions along the Tau sequence, confirmed proximities of the N- and C-terminal domains with the central region of Tau (Mukrasch et al., 2009). Several co-factors were reported to enhance Tau structuration including ions such as Zinc (Roman et al., 2019), potentially regulating Tau functions.

## Map Function of Tau

MT dynamics are regulated by proteins that stabilize or destabilize them (van der Vaart et al., 2009). The main role of Tau, one of the stabilizing MAPs, is to protect MTs against depolymerization by decreasing the dissociation of tubulin at both MT ends, resulting in an increased growth rate and decreased catastrophe frequency (Murphy et al., 1977; Trinczek et al., 1995). *In vitro*, Tau induces MT formation at 37°C and tubulin rings at 20°C under experimental conditions with no self-assembly of tubulin alone, suggesting that Tau is a MT inducer in addition to being a MT stabilizer (Weingarten et al., 1975; Devred et al., 2004; Kutter et al., 2016).

Tau was proposed to bind preferentially to the GDP-tubulin from the lattice, in detriment of the GTP-tubulin from the plus-end cap, and in agreement with a stabilizing role of Tau (Duan et al., 2017). However, monitoring of Tau binding to MTs by FRET has shown Tau decoration on MT tips, as well as on the lattice (Breuzard et al., 2013). Furthermore, the capacity of Tau to induce *in vitro* MT formation is lost when GDP-tubulin is used (Devred et al., 2004). In agreement, Tau depletion in rat cortical neurons results in the loss of MT mass in the axon, predominantly from the labile domain containing tyrosinated tubulin, rather than the stable domain of MTs (Qiang et al., 2018). Green fluorescent protein (GFP)-tagged Tau was indeed observed to be more abundant on the labile domain of the MTs, or GTP-tubulin cap. Based on these observations obtained by quantitative immunofluorescence, the authors propose that Tau is not strictly a MT stabilizer, but rather allows the MTs to conserve long labile domains (Qiang et al., 2018; Baas and Qiang, 2019). However, a more likely explanation is that Tau is necessary to induce tubulin polymerization in the labile region.

Similar to many other IDPs, Tau can undergo phase-transition under *in vitro* conditions of molecular crowding, resulting in the formation of Tau liquid droplets similar to non-membrane compartments (Hernández-Vega et al., 2017). These Tau-rich drops have the capacity to concentrate tubulin 10 times or higher than the surrounding solution. Nucleation of MTs is observed inside the Tau drops, and MT bundle formation results in the elongation of the drops. This bundle formation could not be reproduced by mimicking the high concentration effect, in the absence of drops. This nucleation environment could support stabilization and provide sufficient plasticity for the formation of the long axonal MT bundles. Although it is not yet known whether this phenomenon is of physiological relevance, GFP-Tau droplets have been observed in transfected neurons (Wegmann et al., 2018).

## Structural Aspects of Tau MTBR Interaction With MTs

Tau structure when bound to MTs has been the object of numerous investigations, leading to several models of Tau/MTs complex. *In vitro*, single-molecule FRET experiments showed that Tau bound to soluble tubulin adopts an open conformation (Melo et al., 2016), losing the global fold observed for Tau in solution (Jeganathan et al., 2006; Mukrasch et al., 2009; Schwalbe et al., 2014). However, FRET experiments in the cell, using a Tau protein labeled at its N- and C-termini with fluorophores (Di Primio et al., 2017) show that labeled-Tau bound to MTs displays a global folding, with the N- and C-termini in close proximity, as described for the “paperclip conformation” of free Tau in *in vitro* conditions (Jeganathan et al., 2006). This global fold is lost for unbound Tau, detached from the MTs. This discrepancy might be due to the fact that the cytoplasmic environment is much more complex than can be reproduced in *in vitro* experiments, and Tau might thus bind numerous other cellular partners that could influence its conformation (Di Primio et al., 2017).

Globally, tubulin-bound Tau retains its intrinsically disordered character, and forms a fuzzy complex with tubulin, which might be the reason why Tau binding mode accommodates more than one tubulin (Tompa and Fuxreiter, 2008; Martinho et al., 2018). In agreement, a combination of metal shadowing and cryo-electron microscopy (cryo-EM) revealed that Tau is randomly distributed on the MT surface (Santarella et al., 2004). FRET and fluorescence recovery after photo-bleaching in live cells similarly concluded to the distribution of the Tau/MTs interaction along the MTs, characterized by Tau diffusion coupled to binding phases (Breuzard et al., 2013). In-line with these observations, Tau was shown to diffuse along the MTs, bi-directionally and independently from active transport, in a manner described as kiss-and-hop interactions (Hinrichs et al., 2012). These interactions, observed by single molecule tracking in neurons, might explain why Tau does not seem to interfere with motor protein-mediated axonal transport along the MTs (Janning et al., 2014).

**Figure 3:**
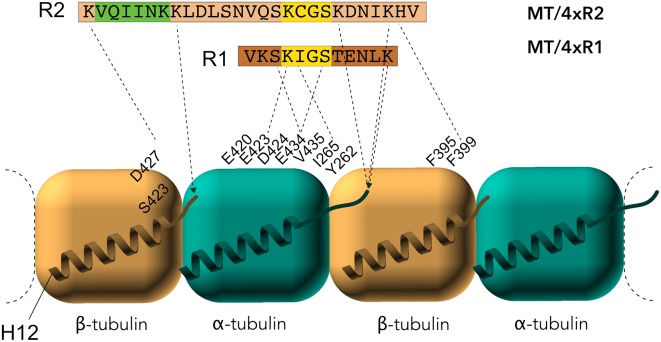
Electron microscopy (EM) models of Tau/MTs interaction. α-tubulin subunit is represented as a blue cube and β-tubulin subunit as a brown cube. H12 C-terminal helix is schematized, the C-terminal tail prolongs the H12 helix. Model based on cryo-EM coupled to Rosetta modeling: contacts of the R1 repeat with the α-tubulin subunit, at the inter-dimer interface: S258 and S262 of R1 make hydrogen bonds with E434; K259 of R1 interacts with an acidic patch formed by E420, E423 and D424; I260 of R1 is in a hydrophobic pocket formed by residues I265, V435 and Y262; K267 of R1 is in contact with the acidic C-terminal tail. Additional contacts for the R2 repeat with β-tubulin subunit, at the intra-dimer interface: K274 of R2 interacts with an acidic patch formed by D427 and S423; K281 of R2 is in contact with the acidic C-terminal tail of β-tubulin subunit. The PHF6* peptide (highlighted green) is close to this tail and localize at the intra-dimer interface. Additional contacts for the R2 repeat with α-tubulin subunit: K294 and K298 are in contact with the acidic C-terminal tail of α-tubulin subunit. Finally, H299 of R2 is buried in a cleft formed by residues F395 and F399 of β-tubulin subunit.

Early studies by peptide mapping allowed the first characterization of the interaction as an array of weakly interacting sites, defining the MTBR as the MT binding core, which contains the four highly homologous repeats R1–R4 (Butner and Kirschner, 1991; Goode et al., 2000). Recently, despite the challenges related to the dynamic and fuzzy nature of Tau/MTs interaction described above, the combination of cryo-EM at near-atomic resolution and Rosetta modeling generated models of the interaction. This breakthrough study highlights the crucial residues of Tau MTBR directly mediating the interaction and confirms the longitudinal mode of Tau binding on MTs (Kellogg et al., 2018). The complex used in the study consists of dynamic MTs without stabilizing agents other than Tau. Tau is found along the protofilaments, following the H11-H12 helices that form a ridge at the MT surface and that are close to the point of attachment of the C-terminal tails of tubulin (Figure 3). The observed density spans Tau residues 242–367, covering the MTBR, and is centered on the α-tubulin subunit while contacts with β-tubulin are detected on both sides. To obtain further details, artificial four R1 and four R2 constructs were used and showed that each repeat of the protein adopts an extended conformation that spans both intra- and inter-dimer interfaces, centered on α-tubulin and connecting three tubulin monomers.

A direct interaction of Tau peptides corresponding to residues 245–253 (in R1), 269–284 (in R2), and 300–313 (in R3) is proposed, based on the attenuation of Tau NMR signal upon addition of paclitaxel-stabilized MTs (Kadavath et al., 2015b). The described bound-motif in the R2 repeat closely matches the bound-stretch found in the EM structure of four R2 Tau repeats, but not the bound motif in the R1, for which the peptide 253–266 is rather proposed as the attachment point for R1 repeat (Kellogg et al., 2018). Additionally, the structure of the bound peptide Tau(267–312; a peptide overlapping R2-R3 repeats) was probed using transfer-NOEs NMR signals. Based on the NOE contacts, which detect spatial proximities, a family of converging conformers were calculated for residues 269–284 (R2) and 300–310 (R3). Both peptides fold into a hairpin-like structure, formed by the conserved PGGG motifs, upon binding to MTs (Kadavath et al., 2015b). However, it should be noted that the electron density in the EM structure models cannot accommodate this hairpin (Kellogg et al., 2018). The hexapeptides 275-VQIINK-280 in R2 (PHF6* or paired helical filament hexapeptide) and 306-VQIVYK-311 in R3 (PHF6; Figure 2) form an extended structure (Kadavath et al., 2015b), in agreement with the EM observation of an extended conformation for R2 (Kellogg et al., 2018). Both the EM and NMR models are incompatible with previous biochemical results, based on combined fluorescence correlation spectroscopy and acrylodan fluorescence screening, suggesting α-helical conformation of the bound-Tau repeats (Li et al., 2015).

The majority of structural data were obtained from the binding of Tau on MTs stabilized by exogenous agents such as Taxol, which facilitates the study by decreasing the dynamics of the system. Still, a number of studies show differences between Tau binding to Taxol pre-stabilized MTs, or to Tau-induced MTs, formed using Tau as an inducer. Kinetics analysis of Tau binding to Taxol-stabilized MTs in comparison with Tau-induced MTs suggests the existence of two different binding sites of Tau to tubulin (Makrides et al., 2004), one overlapping the Taxol binding site localized on β-tubulin in the inner surface of MTs, as previously suggested (Kar et al., 2003).

**Figure 4:**
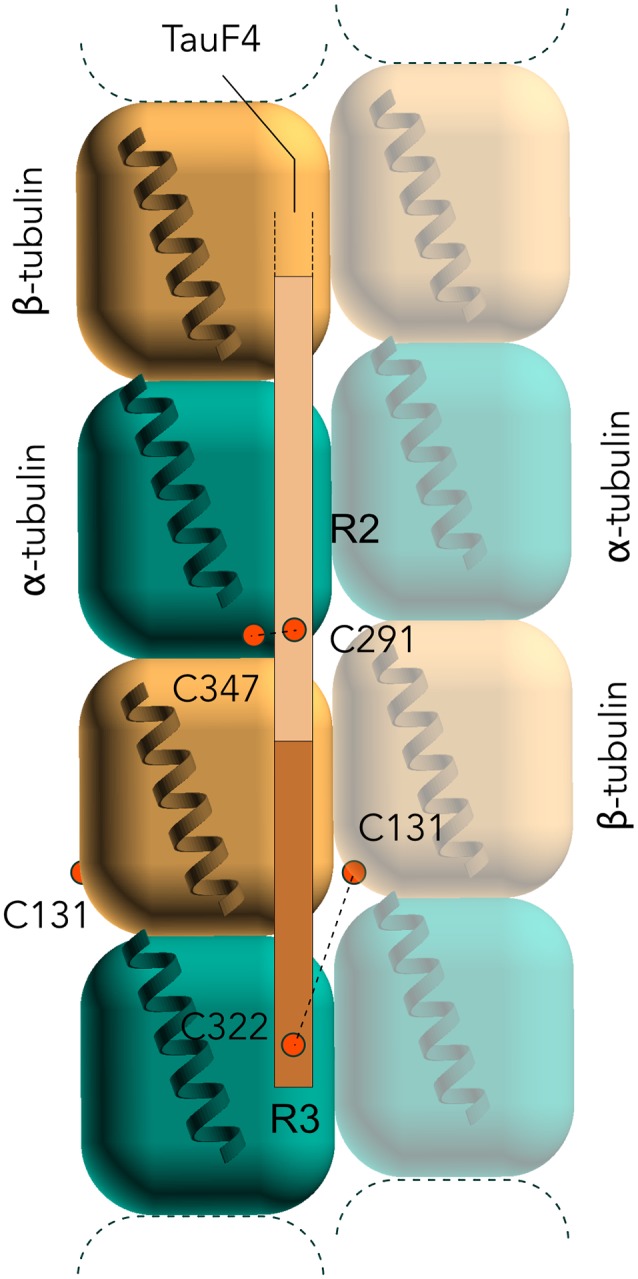
Electron-paramagnetic resonance (EPR) models of the interaction of Tau F4 with MTs. C291 of Tau in R2 is proposed to interact with C347 of α-tubulin, and the C322 of Tau in R3 with C131 of β-subunit. Note that C322 could actually interact either with the β-subunit of the same protofilament or with the one of an adjacent protofilament (as depicted here).

Site-directed spin labeling combined with electron-paramagnetic resonance spectroscopy (EPR) was used to compare Tau binding mode to Taxol-stabilized MTs and to tubulin when Tau is used as the sole inducer of the polymerization (Martinho et al., 2018). In these experiments, thiol-disulfide exchanges between Tau and tubulin or Taxol-stabilized MTs was observed by EPR measurements of paramagnetic labels linked by disulfide bridges to Tau two natural cysteines, or to single-cysteine Tau mutants. Differences in the kinetics of the label release were observed between preformed MTs and Tau-induced MTs, explained by the accessibility of cysteines of tubulin and MTs deduced from available structural data. Localization of the two putative binding sites of Tau on tubulin was proposed (Figure 4). The first one in proximity of Cys347 of α-tubulin subunit could interact with Cys291 of Tau in R2. The second one, located at the interface between two protofilaments, in proximity of Cys131 of β-tubulin subunit, could interact with Cys322 of Tau in R3. The first site is in agreement with models proposing that the R2-R3 would encompass the interface between tubulin (Kadavath et al., 2015a; Kellogg et al., 2018). Both sites are less accessible in Taxol-stabilized MTs, which might explain the much slower release of the disulfide-linked Tau label when Tau is in interaction with the Taxol-stabilized MTs.

## Role of the Regulatory Regions Outside of Tau MTBR

A number of studies addressed the role of the regulatory regions outside the MTBR. The N-terminal projection domain of Tau modulates the formation of MT bundles (Chen et al., 1992; Rosenberg et al., 2008). This domain, described as a polyelectrolyte polymer brush, is proposed based on atomic force microscopy to exert a repulsive force (Mukhopadhyay and Hoh, 2001). In another model, the electrostatic zipper model, Tau is proposed to organize MT spacing by dimerization of the projection domain and the PRR on adjacent MTs (Rosenberg et al., 2008). Both models might be valid but probably depend on the ionic strength of the solution (Donhauser et al., 2017). The N-terminal domain of Tau remains disordered and highly flexible upon tubulin binding, as seen by both NMR (Kadavath et al., 2015a) and smFRET studies (Melo et al., 2016).

Repeats in the MTBR are required for MT binding and assembly. However, as an isolated fragment, the MTBR is not as efficient in tubulin polymerization and in MT binding as full-length Tau. The regions directly flanking the MTBR, in the PRR and in the C-terminal region, are needed to enhance the binding (Figure 2). Several models have been proposed to describe the roles of MTBR and flanking regions. In the first one, termed the “jaws” model (Gustke et al., 1994; Trinczek et al., 1995; Preuss et al., 1997), the PRR, MTBR, and C-terminal extension domains bind very weakly, if at all, to MTs, and the binding is enhanced when two consecutive domains are associated in a single construct. An NMR study has determined the residues in the PRR and in the downstream repeats that constitute the “jaws” (Mukrasch et al., 2007). The second model proposes that the initial binding of Tau to MTs is mediated by an MTs-binding core within the MTBR, whereas the flanking regions are regulatory (Goode et al., 2000).

**Figure 5:**
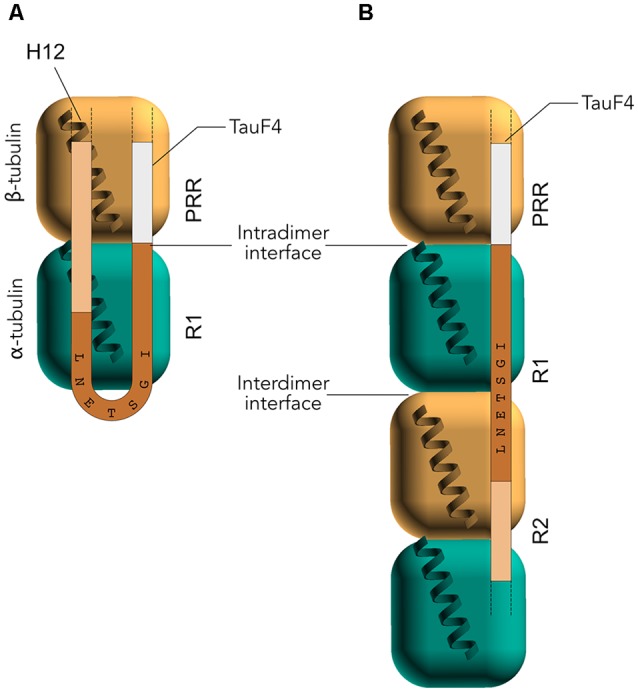
Nuclear magnetic resonance (NMR) model of the interaction of TauF4 with stathmin-like domain (SLD)-stabilized tubulins.** (A)** In interaction with a single tubulin, the IGSTENL peptide of TauF4 is proposed to adopt a turn conformation and is not bound to tubulin. The PRR is mainly detected in proximity to the β-subunit. **(B)** In interaction with two tubulins, the IGSTENL peptide of TauF4 would be straight and overlapping two consecutive α- and β-subunits.

The interaction between full-length Tau and Taxol-stabilized MTs allowed mapping of the binding region on the Tau protein at the residue level (Sillen et al., 2007). In particular, both the MTBR and the flanking regions, namely the PRR and 10 amino acid residues located at the C-terminal end of the MTBR, were found to interact with the MTs. In both the PRR and the MTBR, the contribution of basic residues is important for Tau interaction with MTs (Goode et al., 1997; Kadavath et al., 2015b). Additionally, Tau proteolysis products interacting with tubulin were identified by using a complex named T_2_R composed of two tubulins stabilized by the stathmin-like domain (SLD) of RB3 (Gigant et al., 2000; Fauquant et al., 2011). While the isolated fragments from the PRR and MTBR bind weakly to MTs, the Tau(208–324) construct, named TauF4, generated by combining two adjacent fragments included in the PRR and in the MTBR, binds strongly to MTs and stimulates MT assembly very efficiently (Fauquant et al., 2011). The MTBR included in TauF4 (R1, R2 and the N-terminal part of R3) mostly corresponds to the MT binding core initially proposed by Feinstein and co-workers (Goode et al., 2000). PRE experiments performed by introducing four cysteine mutations on the SLD, located along the protein in proximity of the interface of every tubulin subunit, indicated that TauF4 binds asymmetrically to the two tubulins, with the PRR preferentially located closer to the β tubulin subunits (intra-dimer interface). When bound to a single tubulin stabilized by an engineered SLD protein (TR’), a part of the R1 repeat of TauF4 adopts a turn-like conformation, which remains flexible, and thus not in direct contact with α-tubulin surface (Gigant et al., 2014). The turn-like structure is centered on the 260-IGSTENL-266 sequence (Figure 5). This peptide becomes immobilized in the T2R complex, as can now be explained by its position in the EM structure at the inter-dimer interface (Kellogg et al., 2018). The turn-like structure with a single tubulin present is not detected by smFRET (Melo et al., 2016), which is proposed to result from the different conditions of the experiments (large excess of tubulin for the smFRET or excess of Tau for the NMR experiments). The TauF4 fragment was further shown to bind, at least, at the inter-dimer interface as demonstrated by the competition between vinblastine binding and TauF4 for tubulin interaction (Kadavath et al., 2015a). The results of this study are in agreement with the stoichiometry of one Tau for the two tubulins suggested by others (Gigant et al., 2014).

Finally, the interaction between soluble tubulin and the flanking region downstream of the four Tau repeats was investigated by NMR. This region has sequence homology with the repeats and is often referred to as R’ (Figure 2). By using saturation transfer difference (STD) NMR spectroscopy, two residue stretches centered on F378 and Y394 were identified in interaction with MTs (Kadavath et al., 2018).

## Impact of Tau Phosphorylation on Its Interaction With MTs

Tau-induced neurodegeneration is correlated with the appearance of Tau hyperphosphorylation, Tau aggregation into Paired Helical Filaments and/or the loss of Tau/MT binding (Hernández and Avila, 2007). Overall, phosphorylation is reported to decrease the affinity of Tau for the MTs, ensuring the dynamics of the system in healthy neurons (Lindwall and Cole, 1984; Mandelkow et al., 1995). Specific phosphorylation such as the phosphorylation of S214 (by PKA kinase) or of S262/356 (by MARK kinase) have been described to strongly decrease the affinity of Tau for MTs (Biernat et al., 1993; Scott et al., 1993; Sengupta et al., 1998; Schneider et al., 1999). S262 makes hydrogen bonds with α-tubulin E434, thus explaining why phosphorylation of S262 residue strongly decreases MT binding (Kellogg et al., 2018). Since phosphorylation of S262 of the isolated R1 repeat peptide does not affect its affinity for MTs, it is likely that the consequences of S262 phosphorylation on Tau binding to tubulin are due to intramolecular rearrangements of the Tau protein (Devred et al., 2002). Interestingly, phosphorylation of S214 of Tau does not significantly affect the MT assembly capacity, despite the decreased affinity (Sillen et al., 2007). Phosphorylation of S214 could also have an indirect role *in vivo*, as this specific phosphorylation might play a role in the observed competition between MTs and the 14-3-3 proteins for Tau binding (Hashiguchi et al., 2000). Indeed, 14-3-3 protein binding to Tau is favored when S214 is phosphorylated (Tugaeva et al., 2017), which seems to result in neurite degeneration in neuronal cell cultures (Joo et al., 2015). The 14-3-3 protein family interacts mainly with phosphorylated protein partners and is especially abundant in brain tissue (Boston et al., 1982). The 14-3-3 proteins have been implicated in a variety of human diseases, including neurodegenerative diseases. Indeed, 14-3-3 proteins are abundant in the intraneuronal deposits of aggregated Tau (Layfield et al., 1996; Umahara et al., 2004; Qureshi et al., 2013).

When Tau is phosphorylated by the CDK2/CycA3 kinase *in vitro*, phosphorylation at S202/T205 and T231/S235 sites are identified by NMR. Even though these phosphorylations do not significantly affect Tau binding to MTs (Amniai et al., 2009), Tau loses its capacity to assemble tubulin into MTs when at least three out of four positions are phosphorylated. This data shows that a decreased capacity of Tau to assemble tubulin into MTs, such as observed for the CDK-phosphorylated Tau, cannot be explained solely by a decreased affinity for MT surface. Additional experiments, using the shortest Tau isoform (Tau 0N3R) with T231E and S235E mutations as pseudophosphorylations, confirmed that E231/E235 do not by themselves abolish the interaction of Tau with the MTs (Schwalbe et al., 2015). However, NMR signals corresponding to residues in the PRR were less attenuated upon addition of MTs to the mutated Tau 0N3R rather than to the wild-type Tau (Schwalbe et al., 2015). This indicates that the pseudophosphorylated Tau 0N3R was locally less tightly bound to the MTs. In addition, based on the models of the Tau(225–246) peptide phosphorylated on T231 and S235, issued from comprehensive calculations of NMR parameters (distances measurements and H-N orientations), the distances between the phosphate and the nitrogen in the directly preceding basic groups (R230 or K234, respectively) are less than 4.5Å, a distance compatible with the formation of a salt-bridge. The salt bridge proposed to link phosphorylated T231 and R230 side-chains of Tau could compete with a salt bridge formation with the MTs, participating in the effect of the phosphorylation of T231 (Schwalbe et al., 2015).

Finally, Pin1 peptidyl-prolyl cis/trans isomerase was proposed to restore the capacity of CDK-phosphorylated Tau to bind to the MTs and restore MT assembly (Lu et al., 1999). However, this model was recently challenged as Pin1 does not promote *in vitro* formation of phosphorylated Tau-induced MTs (Kutter et al., 2016).

For repeat R’, the impact of phosphorylation on the binding affinity was assessed by means of STD NMR. Both phosphorylated Y394 and S396 were proved to weaken the interaction between Tau and MTs. However, by measuring residue-specific Kd values by STD, phosphorylation on S396 had a more pronounced effect than phosphorylation on Y394, despite the fact that they are only one residue apart (Kadavath et al., 2018).

Thus, Tau phosphorylation can have an effect on MT binding and assembly through several molecular mechanisms, including electrostatic perturbations, alteration/destabilization of Tau regions bound to the MTs, a disruption of hydrogen-bonds or salt-bridges and changes in structural parameters.

## Impact of Tau Acetylation on Its Interaction With MTs

Another Tau PTM reported to contribute to Tau pathology is lysine acetylation (Min et al., 2010, 2015); indeed, acetylated Tau is proposed as a marker of AD (Irwin et al., 2012, 2013). In this case, Lys residues are modified by the addition of an acetyl group on the NH_3_ moiety of their side chains, neutralizing their positive charges and modifying their steric characteristics. Consequently, acetylation affects Tau binding to MTs and impairs MT assembly (Cohen et al., 2011). Mass spectrometry (MS) analysis revealed that 14 Lys residues, mainly located in the MTBR, were acetylated in Tau samples purified from healthy mice (Morris et al., 2015). Analysis of acetylation sites obtained by *in vitro* acetylation with recombinant p300 acetyl-transferase, by both MS and NMR (Kamah et al., 2014), identified as many as 23 modified-Lys residues. An acetylation mimicking mutation K274Q inhibits Tau tubulin polymerization ability and stimulates Tau aggregation *in vitro* (Rane et al., 2019). Interestingly, acetylation of K280 was detected in brain tissues from patients affected with AD and not in healthy brain tissues (Cohen et al., 2011). Similarly, Tau acetylation at K174 is reported as an early change in AD brain and in transgenic mice expressing Tau with the P301S mutation (PS19 transgenic mice; Min et al., 2015).

In addition, cross-talks between acetylation and phosphorylation modifications of Tau interplay in their regulatory role of MT dynamics (Carlomagno et al., 2017). Acetylation of K321 prevents the phosphorylation of S324, the latter being reported to inhibit Tau function of tubulin polymerization in *in vitro* assays (Carlomagno et al., 2017). Interestingly, this phosphorylation is one of the few modifications of Tau (among 63 modifications) specifically present in a human amyloid precursor protein transgenic AD mouse model when compared to a wild type mouse (Morris et al., 2015). Furthermore, phosphorylation of S324 is detected in post-mortem tissues of AD patients, but not in control samples (Carlomagno et al., 2017). Pseudo-phosphorylated mutants S324D and S324E have a diminished capacity to polymerize MTs from tubulin in *in vitro* assays. Similarly, acetylation of K259/K353 prevents phosphorylation of S262/S356 by the MARK kinase. Modulation of Tau acetylation could be a new strategy to inhibit Tau-mediated neurodegeneration. Indeed, studies in mice suggest that this is a valid disease-modifying target. Increasing acetylation of Tau by deleting SIRT1 deacetylase in a TauP301S transgenic mouse model aggravates synapse loss, while SIRT1 overexpression limits Tau pathology propagation (Min et al., 2018). In the PS19 FTD transgenic mouse model, inhibition of p300 acetyltransferase activity lowers total Tau and K174-acetylated Tau levels. P300 inhibition prevents hippocampal atrophy and rescues memory deficits (Min et al., 2015). However, the complexity of the regulation of Tau function by PTMs has to be kept in mind. Acetylation can have both a direct inhibitory effect on Tau function and an indirect activation effect, by preventing phosphorylation in the KXGS motifs of the MTBR.

## Impact of Tau FTD Mutations on Its Interaction With MTs

Tau protein is encoded by the *MAPT* gene located on chromosome 17. Pathogenic variants in this gene cause several related neurodegenerative diseases characterized by the presence of hyperphosphorylated Tau aggregates in the neurons (Wilhelmsen et al., 1994; D’Souza et al., 1999). Animal models with these mutations, such as P301L, have been extensively studied and are considered AD models (Lee et al., 2005). The FTD mutations of the *MAPT* gene alter Tau biochemical properties and/or the ratio of Tau isoforms (4R/3R ratio). Change in the isoform ratio has an indirect impact on MT assembly and the dynamics of the MT networks because the 3R Tau is known to have a lower capacity of MT stabilization and tubulin polymerization than the 4R Tau (Scott et al., 1991; Panda et al., 2003). The influence on Tau PTMs of the FTD mutations represents another indirect effect on Tau/MT interaction. The R406W Tau mutation is, for example, consistently reported to diminish Tau phosphorylation (Dayanandan et al., 1999; Matsumura et al., 1999; Delobel et al., 2002). However, almost all the FTD mutations also directly affect the ability of Tau to bind MTs and to promote tubulin assembly (Hasegawa et al., 1998; Hong et al., 1998). Tau with P301L, V337M or R406W mutations has a reduced binding to MTs and a decrease efficiency to initiate tubulin assembly. For P301L Tau, the initiation rate is decreased but the tubulin polymerization reaches the same extent in *in vitro* assays. Surprisingly, R406W Tau is reported to be the most affected, despite the fact that the mutation is not near the MTBR (Hong et al., 1998). This suggests that an alternative conformation might be involved. Later studies confirm the initial finding that FTD mutations affect the ability of Tau to bind MTs and to promote tubulin assembly in *in vitro* assays. However, there is no agreement on the extent of the specific effect of each of these mutations (Barghorn et al., 2000; DeTure et al., 2000; Combs and Gamblin, 2012). The effect of R5L, P301L, and R406W mutations of Tau differ in regard to their impact on Tau MT stabilizing capacity, not only based on their localization in Tau sequence, but also depending on the number of N-terminal inserts (0, 1 or 2 N isoforms), in the three considered 4R-Tau isoforms (Mutreja et al., 2019). In particular, Tau with the R5L mutation has a reduced ability to polymerize tubulin, with lower tubulin polymerization extent, lower rate, and longer lag time specifically for the 0N 4R Tau isoform, compared to the 1N and 2N that are behaving as the wild-type Tau (Mutreja et al., 2019). For the P301L mutation, the Tau-dependent defect in MTs seems to diminish with the removal of each N-terminal insert. This might be due to a differential effect of conformational changes induced by the mutations on the global hairpin-like conformation of Tau, which brings the N-terminal region close to the MTBR (Jeganathan et al., 2006). The impact of the FTD mutations on the MT stabilization capacity of Tau has been confirmed in intact cell context, with variable effect depending on the mutation (Delobel et al., 2002). Mammalian cells expressing Tau P301L show proportionally less Tau bound to MTs (Nagiec et al., 2001; Di Primio et al., 2017). However, a strong consensus on the impact of the FTD mutations in a cellular context has not yet been reached, since in neuroblastoma and CHO cells transfected with GFP-tagged Tau DNA constructs, the co-localization with MTs and generation of MT bundles were shown to be identical for both mutants and wild type Tau (DeTure et al., 2000). The site-dependent and isoform-dependent effect of the Tau mutations on MT stabilization were reproduced in COS cells transfected with 3R or 4R Tau isoforms (Sahara et al., 2000). For example, the V337M mutation has a significant effect when introduced in 3R Tau, but not in 4R Tau, showing disruption of the MT networks and diminished co-localization of Tau and tubulin. This isoform-specific effect of some of the mutations might explain part of the discrepancies reported on the impact of the FTD mutations of Tau on its MAP functions.

## Perspectives

One of the proposed strategies in seeking AD treatment consists of compensating the loss of the MT-stabilizing Tau function (Cash et al., 2003; Brunden et al., 2009; Ballatore et al., 2011; Das and Ghosh, 2019). One path to this goal is to harness the therapeutic potential of MT-stabilizing agents, classically used in cancer therapies. This strategy to treat tauopathies was validated *in vivo* using Paclitaxel treatment in AD mouse models (Cash et al., 2003; Zhang B. et al., 2005) and in cell models (Brunden et al., 2011). Recently, Monacelli et al. (2017) provided an updated survey on the potential of repurposing cancer agents for AD. Besides its implication in neurodegenerative disease, Tau is also involved in regulatory mechanisms linked to cancer development. For example, Tau was shown to regulate the MT-dependent migration of cancer cells (Breuzard et al., 2019). Finally, similar to stathmin, Tau level of expression modulates clinical MT-targeting agent efficiencies, such as taxanes or vinca alkaloids (Rouzier et al., 2005; Li et al., 2013; Smoter et al., 2013; Malesinski et al., 2015; Lin et al., 2018).

MT stabilizing peptides are another option chosen to restore MT stability (Quraishe et al., 2016; Mondal et al., 2018). A peptide such as the PS3 octapeptide was designed from the taxol-binding pocket of β-tubulin (Mondal et al., 2018). The advantage of this peptide strategy is the moderate peptide affinity for MTs that preserves the MT dynamic capacity, which is crucial for synaptic plasticity and memory. Consequently, PS3 stimulates MT polymerization and increases expression of acetylated tubulin in PC12 neuron cell cultures but has much fewer toxic effects than taxol. Other neuroprotective peptides have MT stabilizing functions thanks to their ability for interactions with MT end-binding proteins, which protect MT from depolymerization (Oz et al., 2014). The NAP/SAL peptides, which interact with EB1 through a SIP motif, prevent and reverse MT breakdown and axonal transport deficits in a *Drosophila* model of tauopathy (Quraishe et al., 2013, 2016).

Finally, MTs could be stabilized not by mimicking MAP function, but by modulating MT PTMs, which have a crucial role in MT dynamics. Levels of total α-tubulin are reduced by approximately 65% in AD-patient brains compared to age-matched control brains but the relative ratio of acetylated tubulin is increased by approximately 31% compared to the controls (Zhang F. et al., 2015). This suggests a compensatory mechanism to counteract MT reduction due to a loss of Tau stabilization because this modification characterizes stable MTs with slow dynamics (Kull and Sloboda, 2014; Szyk et al., 2014). Inhibition of histone deacetylase 6 (HDAC6), the major tubulin deacetylase, is thus an alternative strategy to compensate for Tau MAP function. Indeed, in transgenic mouse models of AD, inhibition of HDAC6 improves memory (Kilgore et al., 2010; Govindarajan et al., 2013; Selenica et al., 2014). Similarly, in *Drosophila*, HDAC6 null mutation rescues MT defects through increased tubulin acetylation (Xiong et al., 2013).

Overall, Tau implication in neurodegenerative diseases, and other diseases where MTs play an important role, clearly shows the interest of the Tau/MTs interaction as a potential target for intervention (Pachima et al., 2016; Das and Ghosh, 2019). Many advances have been made in the understanding of Tau functions as a MAP, and in the structural aspects of the Tau/MTs interaction. On this basis, the importance of regulatory factors of the interaction, from PTMs to other endogenous cofactors such as Zinc (Fichou et al., 2019), can now be addressed. This will hopefully lead to new strategies targeting disease where MTs, and consequently Tau, are involved.

## Author Contributions

The content of the manuscript was drafted and edited by all authors.

## Conflict of Interest Statement

The authors declare that the research was conducted in the absence of any commercial or financial relationships that could be construed as a potential conflict of interest.

## Abbreviations

AD: Alzheimer’s Disease
EM: Electron Microscopy
EPR: Electron Paramagnetic Resonance spectroscopy
FRET: Förster Resonance Energy Transfer
FTD: Fronto-Temporal Dementia
GFP: Green Fluorescent Protein
HSQC: Heteronuclear Single Quantum Correlation
IDP: Intrinsically Disordered Protein
MAP: Microtubule Associated Protein
MS: Mass Spectrometry
MT: MicroTubule
NOE: Nuclear Overhauser Effect
NMR: Nuclear Magnetic Resonance spectroscopy
PHF6: Paired Helical Filament Hexapeptide
PRE: Paramagnetic Relaxation Enhancement
PTMs: Post-Translational Modifications
SLD: Stathmin-Like Domain
STD: Saturation Transfer Difference.
